# Ringing Up about Breastfeeding: a randomised controlled trial exploring early telephone peer support for breastfeeding (RUBY) – trial protocol

**DOI:** 10.1186/1471-2393-14-177

**Published:** 2014-05-28

**Authors:** Della A Forster, Helen L McLachlan, Mary-Ann Davey, Lisa H Amir, Lisa Gold, Rhonda Small, Kate Mortensen, Anita M Moorhead, Heather A Grimes, Fiona E McLardie-Hore

**Affiliations:** 1 Judith Lumley Centre (formerly Mother & Child Health Research), La Trobe University, 215 Franklin Street, Melbourne, Victoria 3000, Australia; 2 The Royal Women’s Hospital, Grattan St & Flemington Roads, Parkville, Victoria 3052, Australia; 3 School of Nursing & Midwifery, Faculty of Health Sciences, La Trobe University, Bundoora, Victoria 3086, Australia; 4 Deakin Population Health Strategic Research Centre, Deakin University, 221 Burwood Highway, Burwood, Victoria 3125, Australia; 5 Australian Breastfeeding Association, PO Box 4000, Glen Iris, Victoria 3146, Australia; 6 Department of Rural Nursing and Midwifery, La Trobe University Rural Health School, PO Box 199, Bendigo, Victoria 3552, Australia

**Keywords:** Breastfeeding, Exclusive breastfeeding, Breastfeeding rates, Peer support, Telephone, Australia

## Abstract

**Background:**

The risks of not breastfeeding for mother and infant are well established, yet in Australia, although most women initiate breastfeeding many discontinue breastfeeding altogether and few women *exclusively* breastfeed to six months as recommended by the World Health Organization and Australian health authorities. We aim to determine whether proactive telephone peer support during the postnatal period increases the proportion of infants who are breastfed at six months, replicating a trial previously found to be effective in Canada.

**Design/Methods:**

A two arm randomised controlled trial will be conducted, recruiting primiparous women who have recently given birth to a live baby, are proficient in English and are breastfeeding or intending to breastfeed. Women will be recruited in the postnatal wards of three hospitals in Melbourne, Australia and will be randomised to peer support or to ‘usual’ care. All women recruited to the trial will receive usual hospital postnatal care and infant feeding support. For the intervention group, peers will make two telephone calls within the first ten days postpartum, then weekly telephone calls until week twelve, with continued contact until six months postpartum. *Primary aim:* to determine whether postnatal telephone peer support increases the proportion of infants who are breastfed for at least six months. *Hypothesis*: that telephone peer support in the postnatal period will increase the proportion of infants receiving any breast milk at six months by 10% compared with usual care (from 46% to 56%).

Outcome data will be analysed by intention to treat. A supplementary multivariate analysis will be undertaken if there are any baseline differences in the characteristics of women in the two groups which might be associated with the primary outcomes.

**Discussion:**

The costs and health burdens of not breastfeeding fall disproportionately and increasingly on disadvantaged groups. We have therefore deliberately chosen trial sites which have a high proportion of women from disadvantaged backgrounds. This will be the first Australian randomised controlled trial to test the effectiveness and cost effectiveness of proactive peer telephone support for breastfeeding.

**Trial registration:**

Australian and New Zealand Clinical Trials Registry ACTRN12612001024831.

## Background

The risks of not breastfeeding for both mother and infant are well established, yet in Australia, although most women initiate breastfeeding, many discontinue breastfeeding altogether and few women *exclusively* breastfeed to six months as recommended by the World Health Organization and Australian health authorities [1,2]. Infants who are not breastfed have higher rates of gastrointestinal and respiratory illnesses requiring hospitalisation, are more likely to develop Type 1 diabetes in childhood, and have a higher risk of Sudden Infant Death Syndrome, than breastfed infants [3]. Longer term risks of not breastfeeding include higher mean blood pressure and total cholesterol, obesity, higher risk of Type 2 diabetes and lower performance on intelligence testing [4]. Breastfeeding also has health benefits for the mother [5], including a reduced risk of breast and ovarian cancer compared to women who do not breastfeed [3]. Breastfeeding is cost saving for the family and the community [6,7].

The latest national infant feeding survey in Australia, conducted in 2010, found that 96% of children initiated breastfeeding, however only 15% were exclusively breastfed to six months, with 60% receiving any breast milk at six months [8]. In a randomised controlled trial (RCT) by members of the current research group, only eight percent of infants received exclusively breast milk (no solids and no other fluids) to six months [9].

Breastfeeding initiation rates are closely associated with social class, income and education levels in all countries [10]. In Australia, we have reported the widening gap in breastfeeding rates between more and less advantaged women from the 1990s to 2004/2005 [11]. This gap is also clear in the recent national survey: 74% of infants in the highest income quintile are receiving any breast milk at six months, compared to 50% in the lowest income quintile [8].

Breastfeeding rates in Victoria are similar to overall Australian rates [8,12] and also show marked disparities in the proportion of infants receiving *any* breast milk at six months of age in different Local Government Areas (LGAs) around the state [13]. For example, in one Victorian LGA, 68% of infants received *any* breast milk at six months of age, compared with 32% in another [14], highlighting the breastfeeding inequalities between high and low socioeconomic groups. Victorian perinatal data show that term breastfeeding infants from the most deprived socio-economic quintile were more likely to be given infant formula in hospital (26.5%) compared to infants in the least deprived quintile (20.4%; Relative Risk 1.31, 95% CI 1.2, 1.4) [15].

Many women do not reach their intended breastfeeding duration [16], and in our RCT evaluating the effect of an antenatal education intervention to increase breastfeeding, 54% of women who had ceased breastfeeding prior to six months were unhappy with their length of feeding [17]. In another study, 87% of women who ceased breastfeeding within six weeks of birth would have liked to continue longer [18].

The 2012 Cochrane review of interventions that provided support for breastfeeding mothers divided breastfeeding initiation into high (greater than 80%), intermediate (60 to 80%) and low initiation rates [19]. At the proposed trial sites, the Royal Women’s Hospital (the Women’s), Monash Medical Centre (MMC), and Sunshine Hospital (SH), audits in 2009 found that 89%, 91% and 91% of infants (respectively) initiated breastfeeding, and exclusive rates of breastfeeding from birth to discharge were 66%, 78% and 68%. While these figures meet the Cochrane review’s definition of high initiation [19], all three hospitals have a high proportion of women from relatively disadvantaged backgrounds, and local government data from the catchments of these services show average breastfeeding rates at six months of 35% (range 28 to 42%), 12% lower than the statewide average of 47% [20].

### Increasing breastfeeding – evidence from systematic reviews

Evidence on how to maintain breastfeeding in countries such as Australia with intermediate to high breastfeeding initiation is sparse, and most strategies aimed at increasing the duration of breastfeeding have failed. Systematic reviews of strategies to increase breastfeeding have found:

•Antenatal breastfeeding education interventions that increase breastfeeding *initiation* do not increase breastfeeding *duration* as stand-alone strategies [10];

•Breastfeeding promotion interventions may increase breastfeeding in the short term [21], although the increases are generally extremely small, with little significant health impact;

•Breastfeeding education interventions show no association with breastfeeding outcomes [21];

•Extra support (professional *or* lay) increased the duration of breastfeeding (Risk Ratio (RR) for ceasing before six months 0.91; 95% Confidence Interval (CI) 0.88 to 0.96), although there was moderate heterogeneity in included trials; the interventions had a more pronounced effect on exclusive breastfeeding in settings with high breastfeeding initiation [19];

•Lay/peer support interventions increased *any* breastfeeding at six months by 22% (95% CI 8% to 37%), and *exclusive* breastfeeding by 65% (95% CI 3% to 263%) [21]. The Cochrane review found professional and lay support was associated with a positive effect on duration of *any* breastfeeding (RR for stopping any breastfeeding before six months 0.91, 95% CI 0.88 to 0.96) as well as with a positive impact on duration of *exclusive* breastfeeding (RR at six months 0.86, 95% CI 0.82 to 0.91) [19];

•Metaregression analysis of peer support for breastfeeding continuation found that peer support provided solely in the postnatal period was more effective than support provided in both the antenatal and postnatal periods (p < 0.001), and more intensive interventions (at least 5 contacts planned) had a greater effect on breastfeeding continuation than lower intensity interventions (p = 0.02) [22].

### The evidence for peer/lay support as a strategy

“Peer support can be defined as systematic support between two persons or in a group. The participants are regarded as equals . . . A peer supporter is a person who supports breastfeeding, excluding healthcare professionals” [[23] p. 1944]. An alternate definition, from Cindy-Lee Dennis states: “Peer support, within the health care context, is the provision of emotional, appraisal, and informational assistance by a created social network member who possesses experiential knowledge of a specific behaviour or stressor and similar characteristics as the target population, to address a health-related issue of a potentially or actually stressed focal person” [[24] p. 329].

Trials to date of lay (or ‘peer’) support for increasing breastfeeding duration have limited relevance to the Australian context. Those with *positive* results have been mainly in low income countries (Philippines [25], sub-Saharan Africa [26]) and/or countries or communities with low breastfeeding initiation (USA [27-32], Scotland [33]) or high initiation but low exclusivity (Mexico [34], Bangladesh [35]). One trial focused on low birth weight babies in a low income region of Brazil [27]. The results are unlikely to be readily transferable to the Australian context. Other trials did *not* show an effect (in Hong Kong [36], England [37,38] and Scotland [39,40]).

There have been no trials testing peer support for breastfeeding in Australia, and only three internationally that are relevant to our context. An early Canadian trial of telephone support provided by trained volunteers focused on teaching the volunteers about breastfeeding problems, and *had no impact* on breastfeeding duration [41]. An English trial which provided women with access to an existing lay breastfeeding support network was similarly unable to increase breastfeeding duration [37]; although women valued the support they received, the women were unlikely to have been ‘peers’. A Canadian trial implemented *proactive* telephone support by peers who had themselves successfully breastfed (and who were trained to provide support), and achieved a large effect on the proportion of women breastfeeding at three months; 81% compared with 67% in the control group, with no evidence of adverse effects [42].

Breastfeeding is an area of increasing health inequalities, where the costs and health burdens of *not* breastfeeding fall disproportionately (and increasingly) on the more disadvantaged groups [11,43]. The relatively high proportion of women from disadvantaged backgrounds at the proposed sites provide ideal populations in which to trial an intervention to increase breastfeeding.

This will be the first Australian RCT to test the effectiveness and cost effectiveness of a *proactive* approach to peer support for breastfeeding, thus addressing the Cochrane review’s comment that “none of the five studies where women were expected to access support without any proactive element found a difference in outcomes between control and intervention groups” [[19] p.22].

### How does the proposed model differ from existing mother-to-mother breastfeeding support groups?

Mother-to-mother support groups such as the Australian Breastfeeding Association and La Leche League International have provided breastfeeding support for new mothers for about fifty years, a factor associated in time with the marked increase in the proportion of women breastfeeding. However, women who join ABA are more likely to be of higher socioeconomic status (J Lumley, unpublished data). Additionally, organisations such as these rely on women actively seeking support themselves. In our previous RCT of breastfeeding [9] conducted at one of the proposed sites (the Women’s), only 30% of women who said they had breastfeeding problems attended a breastfeeding clinic and 7% contacted ABA. In comparison, a concurrent survey of private patients and family birth centre patients at the same site found that 51% of women with breastfeeding problems attended a breastfeeding clinic and 19% contacted ABA [44]. Women who were public patients were less likely to seek help, especially from existing support groups, than were private patients.

We aim to determine whether peer support, provided during the postnatal period by telephone using a proactive approach, increases the proportion of infants who are breastfed for at least six months.

## Design

A two arm RCT is proposed, recruiting women from three Victorian hospitals whose catchments include areas with some of the lowest breastfeeding rates in the state. Women will be randomised to proactive telephone peer support or to ‘usual’ care.

Our primary hypothesis is that peer support provided to women admitted as public patients by telephone in the postnatal period will increase the proportion of infants receiving any breast milk at six months by 10% compared with standard care (from 46% to 56%).

Secondary hypotheses:

Peer support provided by telephone in the postnatal period will:

a. increase mean breastfeeding duration; and

b. increase exclusive breastfeeding at six months;

We will also evaluate the interventions from the participant and peer support volunteer perspectives; and evaluate the cost-effectiveness of peer support.

### Participants

All eligible women having a baby at the Women’s, MMC and SH during the recruitment period will be offered participation. Women attending these hospitals, although from a wide range of backgrounds, tend to be relatively disadvantaged, with low income and of culturally diverse backgrounds (even among those women who do speak English).

#### Inclusion criteria

Women admitted to the postnatal wards as public patients who have had a first live birth; are proficient in English; and breastfeeding or intending to breastfeed.

#### Exclusion criteria

Serious illness (e.g. severe pre-eclampsia/eclampsia, significant postpartum haemorrhage, severe psychiatric disturbance, pulmonary embolus); infant remaining in hospital after the mother’s postnatal discharge; multiple birth; mother has chosen to formula feed; or antenatal membership of the Australian Breastfeeding Association (ABA), as this may be associated with a higher breastfeeding intention.

### Usual care

All women recruited to the trial will receive usual hospital postnatal care and infant feeding support. The usual length of hospital stay postpartum is two nights following a vaginal birth and three for caesarean births. All women are eligible for one or more home visits by a hospital midwife in the early postnatal period as well as ongoing support from their local Maternal and Child Health (MCH) nurse. Other support needs to be accessed in a proactive manner by women, e.g. breastfeeding clinics (available at all sites and also available in some local government areas) and ABA.

In the state of Victoria, community-based, government-funded support for new parents is provided by the Maternal and Child Health (MCH) Service, a universal primary care service for families with children from birth to school age [45]. The service is provided in partnership with the Municipal Association of Victoria (MAV), Victorian LGAs and the DEECD. The universal MCH Service offers ten consultations to parents (known as Key Ages and Stages (KAS) visits), delivered by Maternal and Child Health Nurses (MCHNs) in MCH centres located throughout all LGAs [45]. MCH centres are located in local communities, often adjacent to kindergartens, and aim to be easily accessible to parents. Victorian MCHNs are registered nurses with additional midwifery and maternal and child health qualifications. The first MCHN consultation takes place at approximately one to two weeks postpartum in the mother’s home. Mothers and infants subsequently attend consultations at their local MCH centre at two, four and eight weeks; four, eight, twelve and eighteen months; and two and three and a half years of age. At each consultation, parents are given the opportunity to discuss concerns, and their child’s health, growth and development. Infant feeding outcomes are collected at KAS visits, with infant feeding practices at hospital discharge, two weeks, three months and six months postpartum reported to the DEECD.

### The intervention

Proactive peer support will be provided by telephone, replicating the intervention found to be effective in the Canadian trial by Dennis et al. [42]. A specific telephone call structure will guide peer contact (see below). Peers will be encouraged to provide most of the contact in the important early weeks, when many women cease breastfeeding, with continued contact tapering off up to six months postpartum. In our previous RCT, 73% of women who were breastfeeding at three months continued until at least six months [9]; we therefore will target the first three months as the critical time for provision of most support.

### Peer volunteers

#### Criteria for peer volunteers

•Lay women who have successfully breastfed for at least six months, who are *not* trained breastfeeding counsellors, but who have a positive attitude to successful breastfeeding.

#### Recruitment of peer volunteers

•Peer volunteers will be recruited from the community by advertisements in local newspapers and pregnancy clinics, distribution of flyers to MCH Centres and by word of mouth. ABA will also advertise for volunteers among members who are not trained breastfeeding counsellors via newsletters and electronic media.

•Women will be asked to ring to express an interest, and will be interviewed/screened for suitability by a member of the research team and or the peer volunteer coordinator.

#### The role of peer volunteers

To provide empathy, encouragement and social support to the women by telephone, as well as to provide information and suggestions about existing clinical and support services (e.g. MCH Nurses, breastfeeding clinics, lactation consultants, General Practitioners, ABA) as indicated and as desired by the participants.

#### Education and support of peer volunteers

•Peer volunteers will undertake education consisting of four hours with an ABA educator. ABA is a Registered Training Organisation and has a short course that they have adapted for training the peer volunteers in this trial.

•The focus will be developing the peers’ skills in listening, information giving, problem-solving, and recognising the need for referral. Strategies for communicating and providing support will be explored, as will the issues of being non-judgemental, empathetic, recognition of boundaries and the need for self-care. Resources in relation to breastfeeding information will be discussed.

•A handbook will be distributed to use, with guidelines for referral and general information.

•Regular ongoing group meetings between volunteers, the volunteer coordinator and investigators will assist with clarifying any issues the peer volunteers may have and to facilitate keeping to the protocol. The volunteer coordinator will also stay in regular telephone and email contact with the volunteers.

•Peer volunteers can contact the volunteer coordinator and trial investigators by telephone at other times for any information, advice or support.

### Contact schedule

*Initial contact*: women allocated to peer support will be telephoned by peer volunteers within four to six days of birth (after discharge from hospital). The *focus of the first call* will be to establish contact, ask how things are going, let the woman know when she will be ‘routinely’ called, and encourage women to ring ‘their’ peer any time they would like someone to talk to, or have a concern regarding breastfeeding.

*Second contact*: the peer volunteer will telephone again three to four days after the initial call (when the baby is eight to 10 days old) to offer: encouragement with breastfeeding; empathetic support regarding adjustment to life with a new baby and the fact that breastfeeding is not always ‘easy’; and to remind women that they are free to ring the peer volunteer whenever they feel it would be helpful (the peer volunteer may also call earlier if they think this will be helpful).

*Frequency of calls*: the peer volunteer will telephone all women at weekly intervals (reduced to two weekly for women who prefer less contact) until the baby is 12 weeks of age. In all cases the focus will be to offer support with breastfeeding in particular, and adjustment to parenthood in general, directing women to existing local services as appropriate or if requested. The peer volunteer will remind each woman of her availability if the woman wants to talk any time between scheduled calls. From three to six months the peer volunteer will continue with less frequent calls (three to four weekly). If women stop breastfeeding, the peer volunteer will discontinue contact.

### Recruitment

Research midwives will recruit women to the trial in the postnatal wards of the study hospitals, at least 24 hours after the birth (unless earlier discharge is planned) and prior to discharge from hospital.

#### Assessment of eligibility

A research midwife will review a computer generated list of all women who have given birth to their first baby in the previous 24 to 48 hours, then approach the staff in the postnatal ward to confirm eligibility.

#### Recruitment and informed consent

The research midwife will follow a protocol to approach women, explain the study, offer trial participation and obtain written consent. It will be made clear that women can withdraw at any time.

### Randomisation

Women will be randomly allocated to peer support or usual care. The randomisation list will be stratified by study site; the randomisation ratio is 1:1 peer support to usual care, with block sizes of four or six distributed randomly. Blocks will be pre-assigned to strata. The total anticipated number of women to be randomised = 1152. Randomisation codes sufficient to allow for recruitment of 1,000 subjects per stratum will be generated.

A computerised system of randomisation designed and administered by an external party will be accessed via the internet to ascertain women’s allocation. The research midwife will follow prompts on the telephone, including entering the woman’s hospital record number. A randomised allocation will be generated, then the woman informed of the outcome.

### Data collection

#### Blinding

The nature of the trial necessitates non-blinding of participants. However, data collection will be undertaken blinded to group allocation where at all possible, recognising that women may volunteer information about having a peer supporter at interview. Data will be presented to the data monitoring committee for the interim analysis in unlabelled study groups. The research team will remain blinded to group allocation until the trial is fully recruited and data cleaning and initial analysis is complete.

#### Data collection

**Figure 1:**
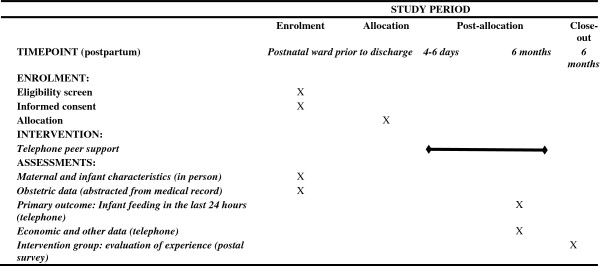
Protocol schedule of enrolment, intervention, and assessments for the RUBY trial participants.

Outcome data will be collected at six months by telephone interview, with baseline data collected at recruitment along with limited obstetric data. The schedule of participant enrollment, intervention and assessments is shown in Figure 1.

### Process and impact evaluation

#### Measures of intervention exposure

Peer volunteers will be asked to keep and regularly submit a log of contacts with their allocated women detailing number and length of calls/visits held with each woman and broad content of discussions in order to assess intervention delivery. This will also enable the volunteer coordinator to follow up if contacts are not occurring as per the protocol. Exposure data will also be collected from the women after completion of their six month telephone interview (see below).

#### Intervention evaluation from the participant and peer support volunteer perspectives

•Women in the intervention group will be sent a short questionnaire to elicit their views about the intervention (after six month data collection);

•When they cease being a peer volunteer the supporters will complete a short questionnaire evaluating their experience of providing support.

#### Cost-effectiveness of peer support

The economic evaluation will first compare the incremental costs and all consequences of the intervention to the control group and then assess cost-effectiveness against any breastfeeding at six months. Data collection for economic evaluation is integrated in the process and outcome evaluation components e.g. household expenditure on infant feeding materials and equipment; health service use since discharge (e.g. admissions, General Practitioner visits, drug treatments, use of midwife/ MCH nurse/other sources of help and advice). Resource use detailed in activity logs will be costed using standard unit costs for telephone expenses and for time use of peers and participants. The trial team will keep detailed records of resources used in peer recruitment, training, support and coordination.

### Sample size

Power calculations for the primary outcome are based on the rate of feeding *any* breast milk in Victoria at six months postpartum. This has been 46 to 47% in recent years (Victorian MCH infant feeding data) with no difference based on whether it is a first or subsequent baby (calculated by DAF using 2008 local government data from three areas) [20]. We estimate a 10% increase to be the smallest clinically important difference that we need power to detect.

An estimated sample size of 822 women based on 80% power (alpha = 0.05) would allow the detection of an increase in the proportion of infants receiving any breast milk at six months from 46% in the control group to 56% in the intervention group (calculated using Stata 9). Allowing 20% loss to follow up, 1028 women are required. This will also provide power to detect a range of other differences (see Table 1). Although the catchment areas of the trial sites show average breastfeeding rates at six months of 35%, it is likely that more motivated women will agree to participate in such a trial, therefore we have taken a conservative approach and based the sample size calculations on the state average. To show a 10% difference from 35% to 45%, a smaller sample size is required, therefore with our current approach we would have more than adequate power to show a 10% difference if the baseline breastfeeding in our sample were 35%. Secondary outcome figures were derived from our previous breastfeeding trial (six month outcomes) [9].

**Table 1:** Power calculations with base number required of n = 411 in each trial arm**

**Outcome**	**Standard care**	**Peer support**	**Power to detect specified difference**
	**%**	**%**	
Primary			
*Any* breast milk at six months	46	56	80
Secondary			
Breast milk *only* at six months^#^	35	45	82

**Allows for loss to follow-up and adjusting for clustering in the analysis.

#Breast milk only type of milk.

To account for potential *within peer* clustering in outcomes for women allocated to peer support we have inflated the sample size by 12% based on simulations to estimate the effect of clustering, assuming an overall average breastfeeding rate of 56% in the intervention arm [46]. We estimated numbers of clusters (individual peer supporters), the average number women in each cluster and a likely range of breastfeeding responses from clusters to calculate an intra-class correlation (rho) of 0.086 and an inflation factor of 1.12. Therefore our final estimated sample size to be recruited is 1152.

### Data analysis

#### Breastfeeding duration

Data will be collected to meet the Consolidated Standards of Reporting Trials (CONSORT) guidelines for reporting of randomised trials [47]. The first stage of analysis will check the comparability of the groups. In relation to the trial hypotheses, the intervention group will be compared to the control group by intention to treat analysis. Proportions of women breastfeeding at hospital discharge and six months will be compared. Duration of breastfeeding (exclusive and partial) will be compared by survival analysis, and the log-rank test used for comparisons. Comparison of means will be undertaken using t-tests where data are normally distributed, or medians compared using Mann–Whitney U tests used if continuous data are not normally distributed. Ranked or Likert type scales will be analysed using Mann–Whitney U tests, and/or cumulative odds ratios. If there are any baseline differences in the characteristics of women in the two groups, which might be associated with the major outcomes, a supplementary multivariate analysis will be carried out.

#### Economic analysis

First stage analysis will be a cost-consequences analysis, with net costs borne by peers, households and health services compared to the above set of primary and secondary outcome measures. Cost-effectiveness analysis will then be conducted against the primary outcome measure (any breastfeeding at six months) to estimate incremental cost-effectiveness ratio in terms of additional cost per additional woman breastfeeding to six months. No discounting will be applied to this one-year evaluation. Extensive sensitivity analysis will be used to explore the impact on cost-effectiveness of uncertainty in cost and outcome data and of possible alternatives to the methodological approach taken (e.g., excluding resource use by households) [48,49].

#### Ethical considerations

Research ethics approval has been obtained from La Trobe University (12–082), Royal Women’s Hospital (12/25), Sunshine Hospital (HREC/12/WH/107) and Monash Medical Centre (12251B). The trial was registered with the Australian and New Zealand Clinical Trials Registry (ACTRN12612001024831) on 24 September 2012.

#### Data Monitoring Committee (DMC)

A DMC will be established to check the randomisation and undertake an interim analysis after 576 women have completed the interview at six months postpartum. The committee of three will include a statistician and a breastfeeding expert with training to participate in a DMC. Criteria will be agreed prior to trial commencement.

### Feasibility

#### Focus group study demonstrating feasibility

In 2006/07 we used focus groups to explore the willingness of women (in the catchment areas of proposed trial sites) to utilise telephone peer support for breastfeeding, and to ascertain what factors would maximise their likelihood of doing so, for example characteristics of the peers, and timing of contact. We also explored methods of recruiting suitable peer volunteers in our community and the willingness of women to act as volunteer peer supporters. We conducted four focus groups including a total of thirty-six women. One group was a targeted group for women from a non-English speaking background. We found:

•*Overall response*: women were positive about the idea of peer support.

•*Contact frequency*: overall there was a sense that this should be individualised and flexible, but relatively frequent e.g. one to two contacts weekly.

•*Preferred peer characteristics*: these were less concerning to women than anticipated; more important was that there was continuity, and that it was someone who had themselves breastfed and who had characteristics such as good listening skills and empathy. Factors such as age and ethnicity were considered less important by women.

•A number of women at each focus group would consider acting as peer volunteers.

#### *Potential uptake and willingness to be randomised*

We undertook a pilot study at RWH in February 2010 to estimate the proportion of eligible women who would be willing to participate in a study of telephone peer support, and of those how many would be willing to be randomised. Of the 189 women potentially available for recruitment (i.e. on postnatal ward), 68 (36%) met the trial eligibility criteria, of whom 58 were approached. Of those women eligible and approached, 39 were willing to take part in a study of peer support, of whom 37 (64% of those eligible and approached) would be willing to be randomised.

The majority of women who would not participate were very supportive of the concept, but did not consider it personally appropriate. Reasons for this included: already having adequate support for breastfeeding, planning to join ABA, or a preference for professional support or ‘hands on’ support.

### Timelines

We expect this trial to take three years. Combined, the trial sites have well over 12,000 births per year, of which approximately 40% will be primiparous.. Assuming an uptake of around 50% based on our feasibility work, and taking into account our eligibility criteria we estimate recruitment will take approximately 15 months. We have allowed 18 months to take into account missing women with short length of postnatal stay, and unexpected periods of non-recruitment e.g. unplanned leave. Following enrolment of the last woman, completion of data collection will take a further six months.

## Discussion

Breastfeeding is an area of increasing health inequalities, where the costs and health burdens of *not* breastfeeding fall disproportionately (and increasingly) on the more disadvantaged groups [11,43]. The relatively high proportion of women from disadvantaged backgrounds at the proposed sites provide appropriate populations in which to trial an intervention to increase breastfeeding. This will be the first Australian RCT to test the effectiveness and cost effectiveness of proactive peer telephone support for breastfeeding.

## Abbreviations

ABA: Australian Breastfeeding Association; CI: Confidence interval; DMC: Data Monitoring Committee; MCH: Maternal and child health; MMC: Monash Medical Centre; NHS: National health survey; RCT: Randomised controlled trial; RR: Risk ratio; RUBY: Ringing Up about Breastfeeding: a randomised controlled trial exploring earlY telephone peer support for breastfeeding; RWH: The Royal Women’s Hospital; SH: Sunshine Hospital; UNICEF: United Nations Children’s Fund; USA: United States of America; WHO: World Health Organization.

## Competing interests

The authors declare that they have no competing interests.

## Authors’ contributions

DAF, HLM, MAD, LHA, RS, LG, KM and AMM conceived the study, developed the protocol and data collection tools, and applied for funding. HG and FMH contributed to design of the trial implementation plan, strategies for recruitment of participants and peer supporters, and to the design and piloting of data collection and process evaluation tools. All authors read and approved the final manuscript.

## Pre-publication history

The pre-publication history for this paper can be accessed here:

http://www.biomedcentral.com/1471-2393/14/177/prepub

## References

[B1] World Health Organization The Optimal Duration of Exclusive Breastfeeding: Report of an Expert Consultation 2001 Geneva: World Health Organization

[B2] National Health and Medical Research Council Infant Feeding Guidelines 2012 Canberra: National Health and Medical Research Council 10.1071/nb05010 16106270

[B3] Ip S Chung M Raman G Chew P Magula N DeVine D Trikalinos T Lau J Breastfeeding and Maternal and Infant Health Outcomes in Developed Countries. Evidence Report/Technology Assessment No 153 2007 Rockville: Agency for Healthcare Research and Quality PMC4781366 17764214

[B4] Horta BL Bahl R Martines JC Victora CG Evidence on the Long-term Effects of Breastfeeding: Systematic Reviews and Meta-analyses 2007 Geneva: World Health Organization

[B5] Labbok MH Effects of breastfeeding on the mother Pediatr Clin North Am 2001 48 1 143 158 10.1016/S0031-3955(05)70290-X 11236722

[B6] Smith JP Thompson JF Ellwood DA Hospital system costs of artificial infant feeding: estimates for the Australian Capital Territory Aust N Z J Public Health 2002 26 6 543 551 10.1111/j.1467-842X.2002.tb00364.x 12530799

[B7] Cattaneo A Ronfani L Burmaz T Quintero-Romero S Macaluso A Di Mario S Infant feeding and cost of health care: a cohort study Acta Paediatr 2006 95 540 546 10.1080/08035250500447936 16825133

[B8] Australian Institute of Health and Welfare 2010 Australian National Infant Feeding Survey: Indicator Results 2011 Canberra: AIHW

[B9] Forster D McLachlan H Lumley J Beanland C Waldenström U Amir L Harris H Dyson K Earl D Two mid-pregnancy interventions to increase the initiation and duration of breastfeeding: a randomized controlled trial Birth 2004 31 3 176 182 10.1111/j.0730-7659.2004.00302.x 15330879

[B10] Dyson L McCormick F Renfrew MJ Interventions for promoting the initiation of breastfeeding Cochrane Database Syst Rev 2005 2 CD001688 10.1002/14651858.CD001688.pub2 15846621

[B11] Amir LH Donath SM Socioeconomic status and rates of breastfeeding in Australia: evidence from three recent national health surveys Med J Aust 2008 189 5 254 256 18759719 10.5694/j.1326-5377.2008.tb02016.x

[B12] Donath S Amir LH Rates of breastfeeding in Australia by State and socio-economic status: evidence from the 1995 National Health Survey J Paediatr Child Health 2000 36 2 164 168 10.1046/j.1440-1754.2000.00486.x 10760016

[B13] Breastfeeding in Victoria: A Reporthttp://www.education.vic.gov.au/healthwellbeing/childyouth/breastfeeding/default.htm

[B14] Maternal and Child Health Services Annual Report (2011–2012) http://www.education.vic.gov.au/Documents/childhood/providers/support/report12.pdf

[B15] Davey M-A Intervention in labour and early breastfeeding outcomes in Victoria, Australia Women Birth 2013 26 S25

[B16] Forster D McLachlan H Lumley J Factors associated with breastfeeding at six months postpartum in a group of Australian women Int Breastfeed J 2006 1 18 10.1186/1746-4358-1-18 17034645 PMC1635041

[B17] Forster DA Breastfeeding – Making a Difference: Predictors, Women’s Views, and Results from a Randomised Controlled Trial PhD thesis 2005 Melbourne: La Trobe University

[B18] Hamlyn B Brooker S Oleinikova K Wands S Infant Feeding 2000 2002 London: The Stationery Office

[B19] Renfrew MJ McCormick FM Wade A Quinn B Dowswell T Support for healthy breastfeeding mothers with healthy term babies Cochrane Database Syst Rev 2012 5 CD001141 10.1002/14651858.CD001141.pub4 PMC3966266 22592675

[B20] Department of Education and Early Childhood Development Maternal & Child Health Services Annual Report 2007–2008 2009 Melbourne: Victorian State Government

[B21] Chung M Raman G Trikalinos T Lau J Ip S Interventions in primary care to promote breastfeeding: an evidence review for the U.S. Preventive Services Task Force Ann Intern Med 2008 149 8 565 582 10.7326/0003-4819-149-8-200810210-00009 18936504

[B22] Jolly K Ingram L Khan KS Deeks JJ Freemantle N MacArthur C Systematic review of peer support for breastfeeding continuation: a meta-regression analysis of the effect of setting, intensity and timing BMJ 2012 344 d8287 10.1136/bmj.d8287 22277543

[B23] Kaunonen M Hannula L Tarkka MT A systematic review of peer support interventions for breastfeeding J Clin Nurs 2012 21 13–14 1943 1954 22672457 10.1111/j.1365-2702.2012.04071.x

[B24] Dennis CL Peer support within a health care context: a concept analysis Int J Nurs Stud 2003 40 3 321 332 10.1016/S0020-7489(02)00092-5 12605954

[B25] Agrasada GV Gustafsson J Kylberg E Ewald U Postnatal peer counsellling on exclusive breastfeeding of low-birthweight infants: a randomized, controlled trial Acta Paediatr 2005 94 8 1109 1115 10.1080/08035250510025752 16188857

[B26] Tylleskar T Jackson D Meda N Engebretsen IM Chopra M Diallo AH Doherty T Ekstrom EC Fadnes LT Goga A Kankasa C Klungsøyr JI Lombard C Nankabirwa V Nankunda JK Van de Perre P Sanders D Shanmugam R Sommerfelt H Wamani H Tumwine JK PROMISE-EBF Study Group Exclusive breastfeeding promotion by peer counsellors in sub-Saharan Africa (PROMISE-EBF): a cluster-randomised trial Lancet 2011 378 9789 420 427 10.1016/S0140-6736(11)60738-1 21752462

[B27] Leite AJ Puccini RF Atalah AN Da Cunha AL Machado MT Effectiveness of home-based peer counselling to promote breastfeeding in the northeast of Brazil: a randomized clinical trial Acta Paediatr 2005 94 741 746 10.1080/08035250410023854 16188778

[B28] Bolton TA Chow T Benton PA Olson BH Characteristics associated with longer breastfeeding duration: an analysis of a peer counseling support program J Hum Lact 2009 25 1 18 27 10.1177/0890334408325985 18971503

[B29] Anderson AK Damio G Young S Chapman DJ Perez-Escamilla R A randomized trial assessing the efficacy of peer counselling on exclusive breastfeeding in a predominantly Latina low-income community Arch Pediatr Adolesc Med 2005 159 9 836 841 10.1001/archpedi.159.9.836 16143742

[B30] Chapman DJ Damio G Young S Perez-Escamilla R Effectiveness of breastfeeding peer counselling in a low-income, predominantly Latina population Arch Pediatr Adolesc Med 2004 158 9 897 902 10.1001/archpedi.158.9.897 15351756

[B31] Pugh LC Milligan RA Frick KD Spatz D Bronner Y Breastfeeding duration, costs, and benefits of a support program for low-income breastfeeding women Birth 2002 29 2 95 100 10.1046/j.1523-536X.2002.00169.x 12000411

[B32] Gross SM Caulfield LA Bentley ME Bronner Y Kessler L Jensen J Paige DM Counseling and motivational videotapes increase duration of breast-feeding in African-American WIC participants who initiate breast-feeding J Am Diet Assoc 1998 98 143 148 10.1016/S0002-8223(98)00037-6 12515413

[B33] Hoddinott P Lee AJ Pill R Effectiveness of a breastfeeding peer coaching intervention in rural Scotland Birth 2006 33 1 27 36 10.1111/j.0730-7659.2006.00071.x 16499529

[B34] Morrow AL Guerrero ML Shults J Calva JJ Lutter C Bravo J Ruiz-Palacios G Morrow RC Butterfoss FD Efficacy of home-based peer counselling to promote exclusive breastfeeding: a randomised controlled trial Lancet 1999 353 9160 1226 1231 10.1016/S0140-6736(98)08037-4 10217083

[B35] Haider R Ashworth A Kabir I Huttly SRA Effect of community-based peer counsellors on exclusive breastfeeding practices in Dhaka, Bangladesh: a randomised controlled trial Lancet 2000 356 9242 1643 1647 10.1016/S0140-6736(00)03159-7 11089824

[B36] Wong EH Nelson EA Choi KC Wong KP Ip C Ho LC Evaluation of a peer counselling programme to sustain breastfeeding practice in Hong Kong Int Breastfeed J 2007 2 12 10.1186/1746-4358-2-12 17883851 PMC2064904

[B37] Graffy J Taylor J Williams A Eldridge S Randomised controlled trial of support from volunteer counsellors for mothers considering breast feeding BMJ 2004 328 7430 26 31 10.1136/bmj.328.7430.26 14703543 PMC313903

[B38] Jolly K Ingram L Freemantle N Khan K Chambers J Hamburger R Brown J Dennis CL Macarthur C Effect of a peer support service on breast-feeding continuation in the UK: a randomised controlled trial Midwifery 2012 28 6 740 745 10.1016/j.midw.2011.08.005 21944571

[B39] Muirhead PE Butcher G Rankin J Munley A The effect of a programme of organised and supervised peer support on the initiation and duration of breastfeeding Br J Gen Pract 2006 56 524 191 197 16536959 PMC1828262

[B40] McInnes RJ Love JG Stone DH Evaluation of a community-based intervention to increase breastfeeding prevalence J Public Health Med 2000 22 2 138 145 10.1093/pubmed/22.2.138 10912550

[B41] Mongeon M Allard R Controlled study of a regular telephone support program given by volunteers on the establishment of breastfeeding Can J Public Health 1995 86 2 124 127 7757891

[B42] Dennis C-L Hodnett E Gallop R Chalmers B The effect of peer support on breast-feeding duration among primiparous women: a randomized controlled trial Can Med Assoc J 2002 166 1 21 28 11800243 PMC99222

[B43] James WPT Nelson M Ralph A Leather S The contribution of nutrition to inequalities in health BMJ 1997 314 1545 1549 10.1136/bmj.314.7093.1545 9183207 PMC2126753

[B44] Amir L Breastfeeding Survey of Frances Perry House and the Family Birth Centre 2002 Melbourne: La Trobe University 1 41

[B45] Maternal and Child Health Service http://www.education.vic.gov.au/childhood/professionals/health/Pages/maternalchildhealth.aspx

[B46] Reading R Harvey I Mclean M Cluster randomised trials in maternal and child health: implications for power and sample size Arch Dis Child 2000 82 1 79 83 10.1136/adc.82.1.79 10630921 PMC1718186

[B47] Schulz KF Altman DG Moher D CONSORT 2010 statement: updated guidelines for reporting parallel group randomised trials PLoS Med 2010 7 3 e1000251 10.1371/journal.pmed.1000251 20352064 PMC2844794

[B48] Forster D McLachlan H Davey M-A Morrow J Newton M Hsueh A Women’s and Staff Views: an Evaluation of Maternity Care at Barwon Health. Baseline Report 2009 Melbourne: Mother and Child Health Research, La Trobe University

[B49] Moher D Schulz KF Altman DG The CONSORT statement: revised recommendations for improving the quality of reports of parallel-group randomised controlled trials Lancet 2001 357 9263 1191 1194 10.1016/S0140-6736(00)04337-3 11323066

